# Ventilation

**Published:** 1883-01

**Authors:** 


### HALL'S JOURNAL OF HEALTH AND MISCELLANY.

MONTHLY.

E. H. GIBBS, A. M., M. D., EDITOR.

FOUNDED IN 1854.

### VENTILATION.

WHEN the air of a room is maintained as nearly as possible at the purity of the external atmosphere, it is well ventilated. In so far as it falls short of this standard it becomes detrimental to health.

The most contaminating ingredient of indoor air is carbonic acid gas; which is poured from the lungs at each expiration, each breath intensifying the impurity, so that the atmosphere of a crowded room or railway car soon becomes nauseating, particularly to a person who enters it from outside. The occupants themselves seldom notice the change that has taken place and generally seem unconscious of the risk to health they are subjecting themselves to.

There are constant emanations of effete matters from the entire surface of the body, and without which life could not be maintained: these add their impurities to the already vitiated air of a close room, thus forming a compound that acts insidiously on the system, contaminating the blood and lessening the power to resist disease. No human being can be subjected to these influences an hour without injury, whether he is conscious of it at the time or not. It is the source from which fevers come; and no doubt other diseases have been conveyed from one person to others under these conditions. If the germs of disease are still active after traveling for a considerable distance through the outer air, how much more potent must they be when inhaled at short distance, warm and fresh from the seat of disease? It is now a well determined fact, that a person with no pre-disposition to consumption is quite liable to contract the disease when exposed to it for some time in an illy ventilated house.

During three years we daily visited some of the hospitals of Paris. Sufferers from small pox occupied the same wards with other fever patients, and no one ever dreamed of taking the disease; but then the ventilation was simply perfect.

The first essential to health is a constant supply of pure, fresh air.

It promotes combustion in the system as a draught of pure air promotes combustion of fuel in a furnace. Carbonic acid gas destroys life; and it will quench a fire sooner than water.

